# Characterization of brain anatomical patterns by comparing region intensity distributions: Applications to the description of Alzheimer's disease

**DOI:** 10.1002/brb3.942

**Published:** 2018-03-06

**Authors:** Diana L. Giraldo, Juan D. García‐Arteaga, Simón Cárdenas‐Robledo, Eduardo Romero

**Affiliations:** ^1^ Computer Imaging and Medical Applications Laboratory – CIM@LAB Universidad Nacional de Colombia Bogotá Colombia; ^2^ Department of Neurology Hospital Universitario Nacional de Colombia Bogotá Colombia

**Keywords:** Alzheimer's disease, ensemble classifiers, MRI, regional analysis

## Abstract

**Purpose:**

This work presents an automatic characterization of the Alzheimer's disease describing the illness as a multidirectional departure from a baseline defining the control state, being these directions determined by a distance between functional‐equivalent anatomical regions.

**Methods:**

After a brain parcellation, a region is described by its histogram of gray levels, and the Earth mover's distance establishes how close or far these regions are. The medoid of the control group is set as the reference and any brain is characterized by its set of distances to this medoid.

**Evaluation:**

This hypothesis was assessed by separating groups of patients with mild Alzheimer's disease and mild cognitive impairment from control subjects, using a subset of the Open Access Series of Imaging Studies (OASIS) database. An additional experiment evaluated the method generalization and consisted in training with the OASIS data and testing with the Minimal Interval Resonance Imaging in Alzheimer's disease (MIRIAD) database.

**Results:**

Classification between controls and patients with AD resulted in an equal error rate of 0.1 (90% of sensitivity and specificity at the same time). The automatic ranking of regions resulting is in strong agreement with those regions described as important in clinical practice. Classification with different databases results in a sensitivity of 85% and a specificity of 91%.

**Conclusions:**

This method automatically finds out a multidimensional expression of the AD, which is directly related to the anatomical changes in specific areas such as the hippocampus, the amygdala, the planum temporale, and thalamus.

## 1 INTRODUCTION

The increased life expectancy and aging of the general population have made of Alzheimer's disease (AD) a growing public health concern. AD is nowadays the most common form of dementia and it is expected that by the year 2050, there will be approximately 135 million patients suffering from different stages of AD (Alzheimer's Disease International, 2013). This means that roughly one in 85 persons worldwide will develop the disease (Brookmeyer, Johnson, Ziegler‐Graham, & Arrighi, 2007), suffer a diminished quality of life, and require constant assistance for the rest of their life.

Despite high human and monetary costs, our AD understanding is at present limited. There is neither a clear disease model nor a plausible physio‐pathological explanation about the underlying progression. In consequence, AD treatment and management are not designed toward planning a personalized therapy or to delay the disease progress but rather to merely treat symptoms, that is, the physician response is purely reactive. AD is commonly diagnosed when a physician detects cognitive impairment or memory complaints, based on the patient's clinical history and a battery of neuropsychological tests measuring different cognitive aspects. A highly variable clinical frame hinders a precise diagnosis which results then fully dependent on the examiner's experience. Only 50% of the cases of probable dementia are correctly diagnosed (Kloppel et al., 2008). The AD diagnosis is confirmed by the detection of plaques and neurofibrillary tangles, possible only by cerebral biopsy or *postmortem*.

The large percentage of misdiagnoses has triggered the interest of the medical community in quantifying the disease degree, a task hardly obtained from the typical neuropsychological tests used in clinics. Under this context, the perspective of using neuroimaging techniques, for both early detection and confirmation, is very appealing and has generated a large body of research. In the clinical practice, however, neuroimaging techniques have had, until now, only a marginal role: Their main use is to exclude other pathological conditions or to visualize the neurodegenerative structural pattern. Recent reviews on the NINCDS‐ADRDA Alzheimer's criteria (Dubois et al., 2007, 2014) recommend using MR neuroimages as a supportive diagnosis tool while the IWG‐2 criteria suggest the use of amyloid PET (Positron Emission Tomography) imaging as evidence of Alzheimer's pathology. Volumetry of the hippocampus and the medial temporal lobe (MTL) has gained wide acceptance as a diagnostic tool and biomarker of the disease progression, yet the disease management is independent of these measures. It should be noted that the presence of atypical AD pathology and other diseases, such as the hippocampal sclerosis, compromises the reliability of the data obtained with this technique.

Quantification of the volume loss and/or structural changes in brain areas has shown an improvement of the differential diagnosis between the AD and some atypical pathological subtypes (Whitwell et al., 2012). Interestingly, this type of quantification does, indeed, resembles the anatomo‐functional correlations that neuroradiologists perform for the early stages of the disease, for example, if there is some evidence of functional impairment, clinicians will search for structural abnormalities and changes in particular brain regions such as the hypothalamus. Although these changes form part of the whole clinical picture, they are usually hard to quantify as they are masked by the huge brain variability, the normal aging process, and the unpredictable progression of the disease.

### 1.1 Pathological and clinical subtypes of AD

Although the disease progression has been traditionally assessed under the Braak and Braak staging scheme (Braak & Braak, 1991), several reports (Akatsu et al., 2002; Armstrong, Nochlin, & Bird, 2000; Janocko et al., 2012; Murray et al., 2014) have demonstrated a very variable AD clinical picture: Neither the progression patterns nor the same anatomical areas are involved or follow a reproducible anatomic sequence, even in series of patients belonging to comparable social and cultural environments. Approximately 25% of AD brains show atypical patterns of structural damage, usually classified as hippocampal sparing and limbic predominant AD (Murray et al., 2011). Furthermore, AD can also manifest different clinical pictures, such as the posterior, logogenic, and frontal variants (IGW‐2). Given that the AD symptoms may be confused with other conditions, there is a strong interest in developing objective tools that characterize the disease progression as well as the clinical variants. Due to their noninvasive and harmless nature, neuroimages constitute a potential source of information. Their utility, however, remains limited as mentioned earlier. Recently, a large number of computational techniques have been proposed, aiming at quantitatively analyzing the radiological information. Aside from the computational requirements, most of these techniques produce binary outcomes, of the type of a disease–healthy classification. Such a categorical labeling is generally not very useful from a clinical standpoint. It should be noted, however, that some groups have developed techniques to assess AD risk deriving continuous metrics from binary classifiers (Casanova et al., 2013; Davatzikos, Xu, An, Fan, & Resnick, 2009; Vemuri et al., 2008).

Despite this, the common radiological analysis is still a structural inspection followed by an abnormality detection which is hardly complemented by these automatic techniques. Most of these methods do not directly quantify radiological observations and physicians end up using their experience as the basis of the diagnosis process. An accurate quantification of radiological findings then turns out to be a strong limitation for integrating these techniques to the radiological analysis workflow. A simple geometrical measurement is apparently inadequate as it excludes the inherent biological variability that hinders the real structural differences induced by the clinical picture.

### 1.2 Computational brain morphometry

Several studies report the use of sophisticated measurement techniques that assess anatomical changes in areas compromised by AD such as the cortical thickness or volumes of subcortical structures (Desikan et al., 2009; Van der Kouwe, Benner, Salat, & Fischl, 2008). The collection of methods that search these anatomical changes or other differences between groups of individual is known as brain morphometry. The brain morphometrical studies are divided into two main steps (Mietchen & Gaser, 2009):

- A common spatial representation of the brain that reduces the inherent anatomical variability among subjects.
- A corresponding morphometrical measure and statistical analysis.

Voxel‐based morphometry (VBM), proposed by Ashburner and Friston (Ashburner & Friston, 2000), is by far the most common approach used by the neuroscience research community. Other features besides the voxel values have been considered in morphometrical studies, giving place to specific techniques such as landmark‐based morphometry (DeQuardo et al., 1999), deformation‐based or tensor‐based morphometry (Ashburner et al., 1998), and surface‐based morphometry (Pantazis, Leahy, Nichols, & Styner, 2004).

These morphometrical analyses require an accurate intersubject registration that guarantees the comparison of homologous structures across all subjects. However, this kind of one‐to‐one correspondence between subjects may not always be achieved, mainly because of the inherent intersubject anatomical variability and the effects of brain pathologies. 1.3 Machine learning techniques.

The use of machine learning and elaborate data processing techniques has become common for the analysis of neuroimages. These approaches train a classifier with different sets of features and assign a label to unseen brain volumes (Davatzikos, Fan, Wu, Shen, & Resnick, 2008; Freeborough et al., 1998). A recent review (Rathore, Habes, Iftikhar, Shacklett, & Davatzikos, 2017) established three automatic classification categories based on the feature extraction method for structural MRI: density maps‐based, cortical surface‐based, and predefined region‐based. As pointed out by these authors, most investigations in the latter category use only hippocampus features as changes in this region are quite systematic, but these studies usually neglect subtle changes or complex anatomic patterns compromising multiple regions. The use of a particular machine learning approach should take this into consideration, that is to say, the algorithm should make hidden patterns to emerge.

Support vector machines (SVM) are the typical automatic classifiers, a technique that assumes there exists a feature space in which two classes are linearly separable. The use of SVM in neuroimaging research has been reported in multiple (Cuingnet, Chupin, Benali, & Colliot, 2010; Cuingnet et al., 2011; Kloppel et al., 2008; Magnin et al., 2009; Padilla et al., 2012; Tanoori, Azimifar, Shakibafar, & Katebi, 2011; Zhang, Shen, & Alzheimer's Disease Neuroimaging Initiative, 2012).

The ensemble of classifiers is an alternative strategy that combines sets of weak classifiers to generate a strong classifier. It has been suggested that given the data high dimensionality and the small size of the available datasets, this ensemble of classifiers shows a better performance than global classifiers (Liu, Zhang, & Shen, 2012). This makes this technique more suitable for the present investigation.

### 1.3 The interpretation gap

Despite the large body of research devoted to propose automatic methods that classify structural MRI cases, there is no so far an accepted standard technique in the clinical practice as in general, for most automatic morphometry techniques, brain areas that express differences are not always correlated with anatomical regions with functional meaning. In case of machine learning approaches, the obtained features usually have no biological interpretability, and despite their good classification results, their contribution to some understanding of the disease progression remains limited. Overall, a main drawback in physio‐pathological terms is that they do compare brains, but their notion of distance has not meaning in terms of the disease progression. Furthermore, in structural terms, these methods may constitute a support to the diagnosis but not a tool for exploration of a spectrum of differences.

### 1.4 Proposed approach

This paper introduces a structural metric that allowed us to estimate brain differences by comparing the intensity levels of functional brain regions, herein understood as a set of contiguous anatomical regions sharing similar functions. The hypothesis underlying this approach is that the subtle neurological differences between AD and NC are correlated with tissue constituents with particular cognitive functions, a feature mirrored by the composition of gray level intensities in the MRI.

This proposal incorporates three guiding principles:

1. The patients do not follow a single unique direction when transitioning from NC to AD. Instead, each patient with AD is assumed to drift away from a healthy state in a particular direction, that is, NC cases form a relatively compact cluster, whereas AD tends to separate toward more than one distinct pathological states.
2. An anatomo‐functional correlation should be used for patient management. To do this, a method that quantifies differences between contiguous anatomical brain regions with specific functional meaning is proposed. This is achieved at the same level experts work in normal clinical practice.
3. The difference between regions is estimated by a true distance between histograms that effectively quantize the frequency information of a gray scale.

## 2 METHODS

The basis for the method is the quantitative measurement of the anatomical differences between individual regions of the brain. The process can be roughly divided in three stages: brain partitioning, region characterization, and pattern extraction. Additionally, the most significant regions in a classification task reveal the structural pattern of the disease.

### 2.1 The brain parcellation

It is well acknowledged that AD tends to affect particular regions of the brain. Furthermore, typical changes have been locally described in AD, for example, strong changes in hippocampus volume. As the disease turns out to be localized and associated with certain functions, it is then reasonable to expect most important changes are concentrated in these regions.

In this work, the Harvard–Oxford brain atlas (Eickhoff et al., 2005) is used as the basic brain parcellation. The atlas covers 48 cortical regions per hemisphere and 17 subcortical structural areas, giving a total of 113 regions. The brain volumes are registered to the atlas by an affine transformation calculated using the FSL (FMRIB Software Library) linear registration tool Flirt (Jenkinson, Bannister, Brady, & Smith, 2002; Jenkinson & Smith, 2001). This registration approach obviously results in slightly displaced intersubject anatomic regions, yet it is herein assumed this is not relevant in terms of the distribution of gray levels within these regions. Such claim may be supported by the fact that overlap between partitioned brains is at least 97%.^1^

By assuming neighboring regions are associated with similar functions, it is then likely that AD disease compromises sets of contiguous regions rather than delimited ones. The aforementioned displacement should in consequence affect marginally the distribution of low‐level features such as the intensity which is the base of the herein proposed characterization.

### 2.2 Region characterization

Ultimately, the objective of our analysis was to be able to quantify the level of similarity or dissimilarity between two subjects. Furthermore, it is expected that this distance is related to the diagnoses given by the expert neurologists, *that is,* two NC subjects should have a higher similarity between them than an NC and an AD subject.

**Figure 1:**
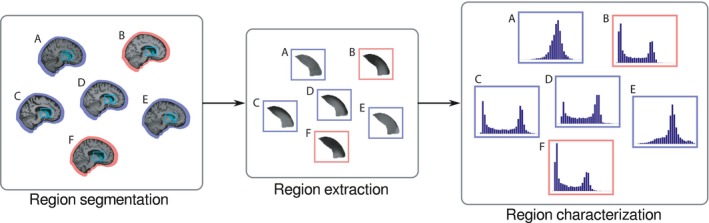
The initial step to recognize patterns is to segment equivalent anatomical regions (left). After the region volumes are extracted (center), further analysis is performed over the grayscale histogram (right)

In this work, the difference between intensity histograms of each anatomical region is measured (Figure 1). These histograms may be interpreted as the probability distributions of the neuronal density of each region as intensities are directly related with the composition of the particular tissue.

#### 2.2.1 Region similarity via Earth Mover’s Distance

Histogram comparison is a common task in many computer vision‐related problems such as image retrieval, color analysis, or 3D object recognition (Ling & Okada, 2007). Although a bin‐to‐bin distance, such as the Kullback–Leibler Divergence (KL) or the chi‐squared distance, is widely used, these measurements are very sensitive to slight region misalignments. This has been overcome by the Earth mover's distance (EMD) (Rubner, Tomasi, & Guibas, 1998), an actual metric between probability distributions that compares how the probability mass of two histograms is distributed along the range of a random variable. Before any comparison, all histograms are shifted so that the center of mass is aligned to the central bin of the histogram. This procedure aims to eliminate the differences by the constant background level when comparing regions.

**Figure 2:**
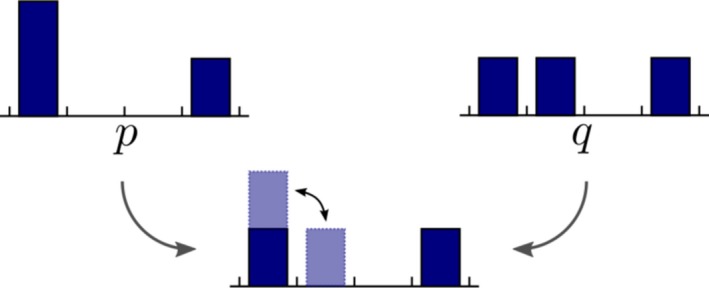
In this case, the EMD between *p* and *q* is the cost of moving one unit from one bin to the next divided by the total mass: 1/3. Notice that the function is symmetric, *that is,* EMD (*p*,* q*) = EMD (*q*,* p*)

EMD calculates the minimum cost of transforming one histogram into another (Rubner, Tomasi, & Guibas, 2000). The EMD is equivalent to solve a linear optimization problem in which certain units of the *S *= {*S*_1_, …, *S*_*n*_} histogram have to be moved to fill the *m* bins of histogram *C *= {*C*_1_, …, *C*_*m*_}, as illustrated in Figure 2 with a simple example of the calculation of EMD between two histograms *p* and *q*.

The movement of one unit from bin *i* ∊ *S* to bin *j* ∊ *C* has an associated cost *p*_*ij*_. The solution consists in a set of movements ${\left\{x_{\mathrm{ij}}^{*}\right\}}_{i,j=1}^{n,\;m}$ that form *C* and minimize the total movement cost. The optimization problem can be written in terms of the amount of “earth,” in this case units *x*_*ij*_, that is moved from bin *i* ∊ *S* to bin *j* ∊ *C*, as follows:

$$\begin{array}{cc} & \min_{x}\sum_{i=1}^{n}p_{\mathrm{ij}}\;x_{\mathrm{ij}}\\ \text{subject to}: & \sum_{j=1}^{m}\;x_{\mathrm{ij}}\leq S_{i}\;\text{for}\;i\in \left\{1,\;\ldots ,n\right\}\\ & \sum_{i=1}^{n}\;x_{\mathrm{ij}}\geq C_{i}\;\text{for}\;j\in \left\{1,\;\ldots ,\;m\right\}\\ & x_{\mathrm{ij}}\geq 0\;\;\forall ij \end{array}$$

In this case, the cost of moving one unit is set to the absolute distance between bins, *that is, p*_*ij*_ = *|i* – *j* |. Given the solution ${\left\{x_{\mathrm{ij}}^{*}\right\}}_{i,j=1}^{n,\;m}$, the EMD between *S* and *C* is the normalized total cost:

$$\text{EMD}\left(S,\;C\right)=\frac{1}{\sum x_{\mathrm{ij}}^{*}}\sum_{i=1}^{n}\sum_{j=1}^{m}\left|i-j\right|x_{\mathrm{ij}}$$

When the compared histograms have the same integral, as in this paper, the problem is symmetric and the EMD is a metric equivalent to the Wasserstein's distance.

### 2.3 Population metrics

The presented pipeline calculates distances between sets of regions from any pair of subjects. However, comparison between regions requires a (unknown) reference. As there is not an explicit representation of the data “center of mass,” the medoid of the control group has been chosen as this center. The medoid is defined as the element of a set with the minimal mean distance to the other population members, *that is,* for a given set *A* and a distance function δ the medoid *x* is defined as:

$$\text{medoid}\left(S\right)=\arg\min_{x\in A}\sum_{y\in A}\delta \left(x,y\right)$$

### 2.4 Region selection

As previously mentioned in Section 2.1, AD does not affect equally all the brain regions. It is important then to sift regions that might contain redundant, misleading, or confusing information for the final analysis. Unbiasedly speaking, the importance of the regions was quantitatively assessed by measuring their usefulness in two classification tasks: separation of patients with Alzheimer's disease and mild cognitive impairment from control subjects.

The most relevant regions are set by an ADABoost (Adaptative Boost) classifier (Freund & Schapire, 1997) trained with the distances of each region to the CN medoid. The ADABoost is an ensemble of meta‐algorithms that iteratively updates the weights of various weak classifiers, giving more importance to samples misclassified in earlier rounds.

Simple thresholds of the distance to the CN medoid have been used as weak classifiers. As there are considerably more CN than AD subjects, the RUSBoost strategy is used (Seiffert, Khoshgoftaar, Van Hulse, & Napolitano, 2010) (Random Unit Sampling Boost), a variation of ADABoost that takes into account the class imbalances by randomly sampling the input during the training step. Once the classifiers have been trained, it is possible to calculate the relative importance of each feature as the weighted sum of mislabeled classes for each predictor.

## 3 EXPERIMENTAL SETUP

The region extracting method was assessed using a subset of the OASIS database (Marcus et al., 2007). The group consisted of 136 brain MR cases, from which 66 were the control group (CN), 50 corresponded to mild cognitive impairment (MCI), and 20 were patients diagnosed with mild Alzheimer's disease. All patients were right‐handed. The age range of both groups was 60–80 years and the distribution by gender is shown in Table 1.

**Table 1:** Age and gender of subjects in OASIS subset

Group	*N*	Age	Gender (F/M)	CDR	MMSE
CN	66	70.76 ± 5.58	48/18	0	29.12 ± 1.12
MCI	50	72.80 ± 5.03	28/22	0.5	26.04 ± 3.49
AD	20	74.30 ± 4.33	13/20	1	20.75 ± 3.65

AD, Alzheimer's disease; MCI, mild cognitive impairment; OASIS, Open Access Series of Imaging Studies; CDR, Clinical Dementia Rating; MMSE, Mini‐mental State Examination.

The brain volumes in the database were acquired with 1.5 T Vision scanners (Siemens, Erlangen, Germany), using T1‐weighted magnetization‐prepared rapid gradient‐echo (MP‐RAGE) sequences. Images were spatially warped into the 1988 atlas space of Talairach and Tournoux (Buckner et al., 2004), averaged, skull‐stripped, and finally, gain‐field corrected to obtain a single, high‐contrast MP‐RAGE image per subject. For more detailed information about this process, see Marcus et al. (2007).

Classification performance was assessed via a receiver operating characteristic curve (ROC). Due to the limited size of the dataset, the ROC was calculated using a leave‐one‐out scheme, *that is,* iteratively training with the whole set but one and then using the resulting classifier to classify the case set aside.

### 3.1 Testing different populations

Generalization of the presented method was tested by performing the analysis in an independent database, an issue commonly avoided when assessing automated neuroimaging methods. Specifically, the analysis was carried out with the MIRIAD database (Malone et al., 2013) using the classifier trained with the OASIS database.

The MIRIAD database is composed of 69 brain MR images from 23 healthy controls and 46 subjects diagnosed with probable Alzheimer's disease. As described in (Malone et al., 2013), images were acquired with a 1.5 T Signa MRI scanner (GE Medical systems, Milwaukee, WI, USA), using a T1‐weighted inversion recovery‐prepared fast spoiled gradient recalled (IR‐FSPGR) sequence. Images were warped into the Talairach and Tournoux atlas and skull‐stripped using the FSL (Jenkinson, Beckmann, Behrens, Woolrich, & Smith, 2012) package. The distribution of age, gender, and clinical scores of the used data is presented in Table 2.

**Table 2:** MIRIAD database subject distribution

Group	*N*	Age	Gender (F/M)	CDR	MMSE
CN	23	69.67 ± 7.06	11/12	0	29.39 ± 0.84
AD	46	69.34 ± 7.18	27/19	1.01 ± 0.36	19.20 ± 4.01

AD, Alzheimer's disease; MIRIAD, Minimal Interval Resonance Imaging in Alzheimer's disease.

The difference measurements have been implemented using MATLAB (2010), running on a Linux PC with 2 Intel Quad Core i7 at 3.07 GHz and 24 GB of RAM. The most time‐consuming part of the algorithm is the calculation of the EMD, which takes in average 6 s for 64‐bin histograms.

## 4 RESULTS

### 4.1 Classification results

**Figure 3:**
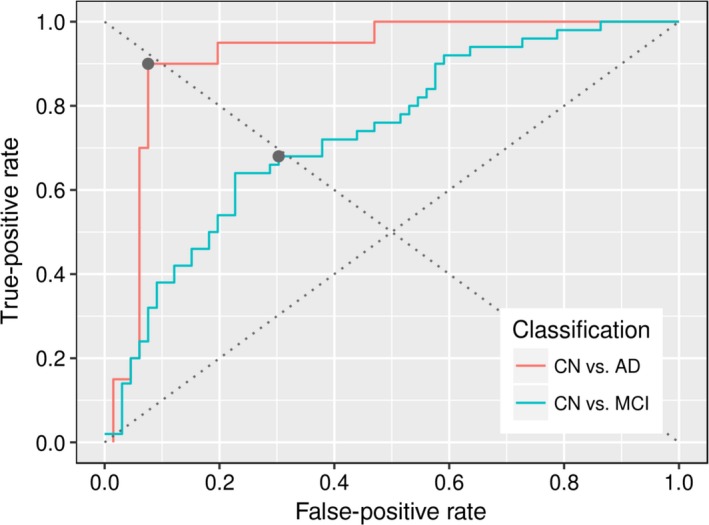
Receiver operating characteristic curves for both experiments with OASIS database, the gray dots correspond to the classification instances where the two types of error are equal

The resulting ROC curves are shown in Figure 3, from which two common classification performance measurements are computed, the area under the curve (AUC) and the equal error rate (EER), being this last measure the threshold point at which false‐positive (FP) and false‐negatives (FN) type errors are equal. The EER is 0.1 and the AUC is 0.92 when classifying between CN and AD cases of the OASIS dataset, while classification between CN and MCI shows the EER is 0.3 and the AUC is 0.74. By comparison, previous works performing a classification between CN and AD with the same experimental setup obtained an EER of 0.86 (Rueda, Gonzalez, & Romero, 2014) and 0.8 (Toews, Wells, Collins, & Arbel, 2010) (AUC was not reported), *that is,* the present strategy outperforms both methods.

### 4.2 Interpopulation results

The classifier trained with the OASIS database was assessed for the MIRIAD database, aiming to test the generalization of this method. This experiment resulted in a sensitivity of 85% and specificity of 91%. These results show a good overall performance with a high accuracy. The errors consist mostly of false positives (seven cases), whereas the number of false negatives remains relatively low (two cases).

### 4.3 Region selection

As RUSBoost uses random sampling, the most relevant regions were selected by averaging the importance of the regions for the total number of iterations of the validation scheme. The Harvard–Oxford Atlas segmentation, with 96 cortical regions (48 per hemisphere) and 17 subcortical regions, was used for a coarse extraction of regions. The most relevant regions to discern between controls, AD, and MCI patients are shown in Tables 3 and 4, respectively.

**Table 3:** Top 10 most relevant regions for the classification between control subjects and patients with Alzheimer's disease (AD)

	Region	Importance (%)
Right	Hippocampus	15.51
Right	Planum temporale	13.35
Left	Hippocampus	8.40
Left	Thalamus	7.16
Right	Paracingulate gyrus	4.83
Right	Middle temporal gyrus, anterior division	4.38
Left	Insular cortex	4.17
Right	Putamen	3.59
Left	Front orbital cortex	2.71
Right	Amygdala	2.39

**Table 4:** Top 10 most relevant regions for the classification between control subjects and patients with mild cognitive impairment

	Region	Importance (%)
Left	Amygdala	6.35
Right	Hippocampus	5.41
Left	Hippocampus	4.78
Right	Planum temporale	3.56
Right	Heschl's gyrus	3.24
Left	Inferior frontal gyrus, pars triangularis	3.10
Right	Middle temporal gyrus, anterior division	2.95
Right	Amygdala	2.55
Left	Paracingulate gyrus	2.40
Left	Parahippocampal gyrus, anterior division	2.18

Inspecting the regions ordered by relevance, it is evident that, in the case of CN/AD classification, the discerning power is concentrated in few regions: The top 10 most relevant features summed more than 66.5% of the relevancy and the most relevant region (right hippocampus) amounts to a relevancy of 15.51%.

Provided that anatomical changes by MCI are not expected to be so marked, differences are subtle and more regions need to be taken into account to detect them, in other words, importance is more spread across the regions and patterns turn out to be more complex.

**Figure 4:**
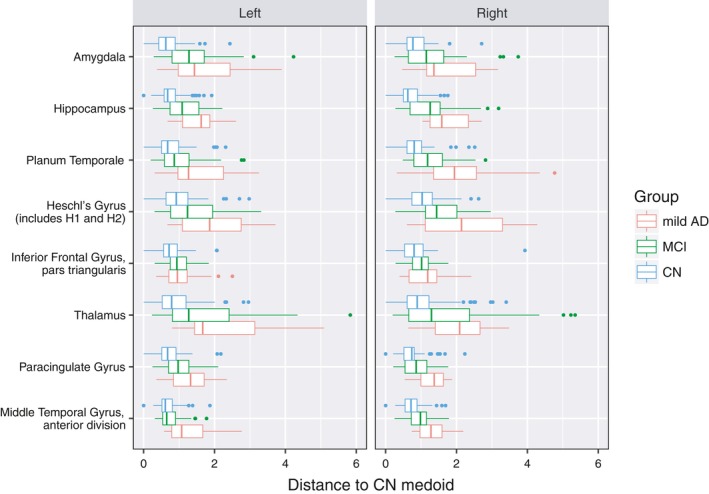
Results of the distances to the medoid for the most relevant regions together with their contralateral equivalent

It is worthy to mention that relevance is mostly “additive” that is to say a region with little relevancy is in any case informative, but this information is redundant and shared by other regions. In this case, the classifier will assign relevance to those regions that add value to the classification task. This statement is illustrated by the distributions of the two hippocampi in Figure 4: Although both regions show similar distribution trends and strong interclass separation, the right hippocampus is more relevant than the left one, which shows almost half of the importance in Table 3. It is likely that this difference reflects a cerebral dominance trend. This difference appears during the classifier training phase: The weak classifier, based on the left hippocampus, mostly confirms the results of its right counterpart, that is, as the same cases are discriminated by both left and right weak classifiers, RUSBoost considers the former redundant as not much additional information is added by it and its weight is decreased in the ensemble of classifiers. Because of this, regions showing important changes along the whole population are ranked higher, whereas those regions useful to classify particular cases are ranked lower.

When analyzing the box plot graphic of the distances to the medoid of the most relevant regions and their opposite hemisphere equivalences (shown in Figure 4), there are strong observable differences between NC, which form relatively compact groups, and AD subjects, which tend to be more scattered and diverge from NC, while the MCI group falls between them. This trend is particularly remarkable in the amygdala, hippocampus, planum temporale, and thalamus, where the NC and AD distributions are well separated.

A good indicator of the validity of the present methodology is the observation that although all regions are treated equally, without making any a prior assumptions, the regions selected as highly relevant are acknowledged by clinicians as characteristic of the AD changes in terms of diagnosis and evolution, especially the hippocampus, whose role as a biomarker of the disease progression is widely accepted. The present metrics, in addition, quantify different patterns of the disease and allows us to establish a distance between these differences.

## 5 DISCUSSION

This paper introduces a novel automatic strategy that detects characteristic structural brain patterns associated with the presence of the Alzheimer's disease in two public brain MR datasets. The process begins by applying a publicly available registration tool (Jenkinson & Smith, 2001; Jenkinson et al., 2002) to align all volumes to a brain atlas (Eickhoff et al., 2005). The regional intensity distribution differences between subjects are then estimated using the EMD. These intersubject anatomic features were used in a related binary classification task to establish the contribution of each region to the discrimination between healthy and unhealthy individuals. The underlying hypothesis behind this method is that any regional comparison shows the relative loss of neuronal tissue which is the main characteristic of any neurodegenerative disease. The method uses distances between estimations of the intensity distribution, assuming the neural density correlates with the gray levels of the MR image. Some side advantages of the presented methodology are its simplicity, reproducibility, and interpretability.

**Figure 5:**
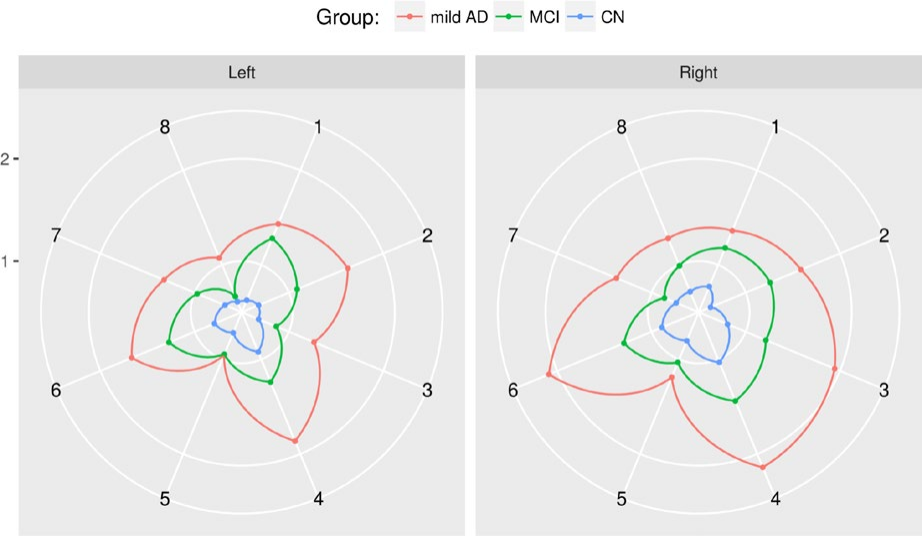
The directions of these polar graphics correspond to the eight most relevant regions in the classification task: 1. amygdala, 2. hippocampus, 3. planum temporale, 4. Heschl's gyrus, 5. inferior frontal gyrus, 6. thalamus, 7. paracingulate gyrus, and 8. middle temporal gyrus (anterior division)

Classical voxel or deformation‐based strategies has been able to establish statistical anatomical intergroup differences, but they show limitations finding the exact compromised brain regions along an experimental group as the analysis considers every voxel independently. Analyses have evolved from these local approaches to the regional concept of identifying scale‐invariant salient features (Toews et al., 2010). These sets of features have been found to be group‐related and suitable biomarkers, but not useful for finding out anatomo‐physiological correlations that enhance the understanding of a particular disease. In contrast, the approach herein described can be seen as an improvement over current methods as is clinically interpretable by standing out actual patterns of the disease. This idea is illustrated in Figure 5 which shows different quantified patterns of the disease using the proposed metrics.

Figure 5 shows the median per group of the distances to the CN medoid for each region. This visualization allows us to observe a progression pattern in the different compromised regions.

This figure also suggests that equivalent regions in the two hemispheres could not show the same progression rate and then the level of discrimination between subjects is better when the left and right equivalent regions are taken separately, a claim corroborated with additional classification experiments in which results deteriorated when the left and right regions were combined (data not shown).

It should be noted that the main goal of the present research was not to simply develop a fully automatic classification process but rather to find a strategy to automatically highlight regions characterizing the disease. Nevertheless, the presented approach did effectively separate the different groups. To put this in context, the classification between controls and patients with AD achieved 90% sensitivity and specificity while the best performing methods out of 10 compared in Cuingnet et al. (2011) using a different database reported up to 81% sensitivity and 95% specificity. Similar or slightly lower results were found for methods relying on tissue segmentation (Davatzikos et al., 2008; Fan, Resnick, Wu, & Davatzikos, 2008; Westman, Aguilar, Muehlboeck, & Simmons, 2013; Zhang, Wang, Zhou, Yuan, & Shen, 2011), elastic deformations (Magnin et al., 2009), semiautomatic segmentation of the hippocampus (Barnes et al., 2004), or combinations of one or more of them (Farhan, Fahiem, & Tauseef, 2014; Kloppel et al., 2008; Plant et al., 2010; Teipel et al., 2007; Wolz et al., 2011).

On the other hand, an issue regarding this methodology is the use of a rigid registration, instead of the usual elastic match performed by most of methods. This decision aims to introduce simplicity and reproducibility. Furthermore, the method attempts to find out those brain areas with intensity differences or neuronal loss, a task for which the anatomical regions may have some displacement as the important measurement is the distribution of gray levels. In the present investigation, the exact quantification of the overlap could only be measured by a set of experts delineating all regions. However, a rough overlapping estimation with the Dice coefficient between the different brains was computed as.

In practice, the high level of agreement seen in Table 5 suggests the overlapped brain area is high enough to guarantee that the distribution of intensities is representative, even when some regions displacements may be present. As measured differences depend on the number of intensities, the results are sufficiently robust to probable small misalignments.

**Table 5:** Dice score by overlapping the different database brains after the rigid registration

	OASIS	MIRIAD
OASIS	0.99 ± 0.0030	0.93 ± 0.0057
MIRIAD	0.93 ± 0.0057	0.96 ± 0.0062

MIRIAD, Minimal Interval Resonance Imaging in Alzheimer's disease; OASIS, Open Access Series of Imaging Studies.

Finally, future work should include evaluation with larger databases to confirm what has been observed in the present study. Likewise, the method should be assessed with different databases, yet a preliminary analysis was carried out in the present investigation using a different database. In such a task, a classifier with no further training found a high level of sensitivity despite the strong gender wise, class imbalance (i.e., the NC to AD ratios) and sample size differences. It is worthy to mention that the two databases, training and testing, were acquired with equipment from different manufacturers with their own proprietary acquisition protocols.

## Note

1 Experiments performed by the authors showed this figure for the OASIS database.
